# Neuropsychiatric symptoms in vascular dementia: Epidemiologic and clinical aspects

**DOI:** 10.1590/1980-57642018dn12-010006

**Published:** 2018

**Authors:** Marcelo Antônio Oliveira Santos, Lucas Soares Bezerra, Carolina da Cunha Correia, Igor Silvestre Bruscky

**Affiliations:** 1 Director of the Epidemiology and Cardiology Research Group, Federal University of Pernambuco, Recife, PE, Brazil. Medical Student at Mauricio de Nassau University, Recife, PE, Brazil; Mauricio de Nassau University, Recife, PE, Brazil; 2 Full Professor of Neurology, Maurício de Nassau University, PE, Brazil; 3 Neurologist at Areias General Hospital Elderly Referral Unit, Recife, PE, Brazil

**Keywords:** vascular dementia, elderly, behavioral symptoms, neuropsychiatric symptoms, demência vascular, idosos, sintomas comportamentais, sintomas neuropsiquiátricos

## Abstract

Dementia is a general term for a heterogeneous group of organic neurodegenerative diseases. Cerebrovascular causes account for 20% of cases.

**Objective:**

To describe the clinical and epidemiological features of individuals aged >60 years diagnosed with vascular dementia (VD) or mixed dementia (MxD) in a referral hospital for dementia.

**Methods:**

A descriptive, retrospective study was carried out from 2014 to 2017 involving elderly individuals (≥60 years) with VA or MxD. Patients presenting other forms of dementia or in use of medication that mimics cognitive disorders were excluded. The 12-item Neuropsychiatric Inventory was used to assess neuropsychiatric symptoms (NPS).

**Results:**

81.1% of the patients presented NPS and only 15% had two or more symptoms. Apathy was the most frequent NPS (56.6%). There was an association between CDR score 1 or 2 and NPS (OR = 6.16, 95% CI: 1.36-27.9, p = 0.02).

**Conclusion:**

Most patients had a single symptom, predominantly apathy. There was an association between mild-to-moderate dementia and NPS.

## RESUMO

Demência é uma terminologia geral para um grupo heterogêneo de doenças orgânicas e neurodegenerativas. As causas cerebrovasculares são responsáveis por 20% dos casos.

**Objetivo:**

Descrever as características clínicas e epidemiológicas de indivíduos maiores de 60 anos diagnosticados com demência vascular ou mista em hospital de referência em demência.

**Métodos:**

Estudo retrospectivo descritivo de 2014 a 2017 em idosos com demência. Outras formas de demência ou uso de medicamentos que mimetizem distúrbios cognitivos foram excluídos. O inventário neuropsiquiátrico de 12 itens foi usado para avaliar os sintomas neuropsiquiátricos (SNP).

**Resultados:**

81.1% dos pacientes apresentaram SNP, apenas 15% tiveram dois ou mais. Apatia foi o mais frequente (56,6%). Houve associação entre CDR 1 ou 2 e SNP (OR = 6.16, 95% CI: 1.36-27.9, p = 0.02).

**Conclusão:**

Geralmente há um sintoma isolado, sendo apatia o principal. Encontramos associação entre demência leve e moderada e SNP.

Dementia is a general term for a heterogeneous group of organic, progressive and impairing brain diseases. The disease is expected to affect approximately 42.3 million people worldwide by the 2020.^1^ Cerebrovascular diseases, which account for at least 20% of dementia cases, are the second most common cause of dementia.^1^^-^ 3 However, combination with other factors involved in dementia physiopathology is not infrequent, a condition referred to as mixed dementia (MxD).^3^ Some studies in Brazil suggest a prevalence of vascular dementia (VaD) ranging from 9.3% to 24.9%.^4^ Even in pure VaD, physiopathology is heterogeneous because several vascular conditions can lead to dementia. Nevertheless, there is a lack of studies on the correlation between clinical presentation and progression in the different subtypes of VaD and NPS.^5^^,^ 6 VaD is associated with atherosclerosis, age, systemic arterial hypertension, hypercholesterolemia, hypertriglyceridemia, diabetes mellitus and other neurovascular risk factors.^7^

Neuropsychiatric symptoms (NPS) are common both in patients with vascular dementia (VaD) and MxD and those with Alzheimer's disease (AD), in which greater severity of dementia is a factor for poor prognosis.^8^ However, there is no consensus on whether NPS are more common in VaD and MxD or in other types of dementia (e.g. AD) due to the variety of different evaluation instruments used to assess neuropsychiatric symptoms.^6^ Currently, the Neuropsychiatric Inventory (NPI) is the most used instrument for evaluating these symptoms, both in research settings and clinical practice. The NPI was originally created to assess 10 NPS commonly seen in dementia and was subsequently modified to evaluate 12 disturbances.^9^ The 12-item NPI has been translated and validated in Brazilian Portuguese.^10^

Agitation, apathy, mood changes, day-night cycle disturbance, hallucinations, and hostile behavior are among the most prominent NPS in patients with dementia. All these symptoms are often present during the late stages of the disease course and can exacerbate patient disabilities and result in high caregiver distress or burnout syndrome as well as a high risk of patient deterioration.^5^^-^ 7 ^,^ 11

Despite the importance of this subject, available data about the neuropsychiatric profile of vascular dementia in the literature is insufficient and few studies reflect circumstances in Brazil. The objective of this study was to describe the clinical and epidemiological features of individuals aged >60 years diagnosed with VaD or MxD according the DSM-V criteria in a referral hospital for diagnosing and treating dementia and cognitive impairments in elderly.

## METHODS

A retrospective, descriptive study was performed from January 2014 to January 2017 at the Elderly Referral Unit of the Areias General Hospital. The study included patients of both gender aged 60 years or over diagnosed with VaD or MxD, based on DSM-V criteria, and followed at the study site. Patients with other dementia types, conditions and medications that could simulate cognitive disturbances were excluded. A total of 86 patients were initially recruited, of which only 53 patients satisfied both inclusion and exclusion criteria.

The study was approved by the Ethics Committee of Maurício de Nassau University (UNINASSAU) under protocol CAAE 68601817.7.0000.5193 and complied with Resolution 196/96 of the National Health Council, which stipulates guidelines and standards for research involving humans.

The Mini-Mental State Examination (MMSE) questionnaire was used to assess global cognitive functioning. The cut-off points were established in accordance with the Brazilian study conducted by Brucki et al.^12^ The Clinical Dementia Rating (CDR) scale was used to rate dementia severity.^13^

Patients underwent a full routine evaluation, including: the MMSE, a battery of cognitive tests, the CDR scale, a neurological examination, laboratory screening tests, and a brain CT. After establishing the diagnosis, the 12-item NPI^9^ was applied to assess behavioral symptoms.

Data was collected by independent researchers through standard forms from medical records and processed using the Statistical Program for Social Sciences (SPSS), version 20.0. A descriptive analysis of clinical and epidemiology characteristics was performed, using non-parametric tests, since none of the variables had a normal distribution . Spearman's correlation analysis was also applied. Chi-square tests were also performed. Age, sex, MMSE score and number of symptoms were each chosen as independent variables for multiple ordinal regression. According to the sample size calculation determined by the OpenEpi version 2 software, 52 patients would suffice for the present study, given a minimum symptom frequency of 56%,^14^ considering an alpha error of 5% and an statistical power of 80%.

## RESULTS

A total of 53 patients were included in this study. The average age was 76.7 ± 9.4 years [mean ± SD]). 34 (64%) subjects were male, with an average disease duration of 7.2 ± 3.6 years, mean CDR score of 1.8 ± 0.7 points and mean MMSE score of 13.9 ± 5.2 points. No statistical differences were found for distribution of dementia etiology, age, sex, disease duration or CDR score in the sample.

The mean age at dementia diagnosis was 68.4 ± 7.4 years. The prevalence of any neuropsychiatric symptoms was 81.1% and only 15% presented two or more symptoms. Apathy was the most frequently encountered neuropsychiatric symptom (56.6%), followed by both irritability and anxiety (18.9% each), and sadness and disinhibition (5.6% each). All sample characteristics are shown in Table 1.

**Table 1:** Distribution of clinical and epidemiological characteristics for the studied sample.

Sample characteristics				Total = 53 n (%)		
**Gender**	Male	34 (64)		**Neuropsychiatric symptoms**	None	10 (18.9)
	Female	19 (36)			Apathy	30 (56.6)
**Age (years)**	60-69	11 (20.7)			Irritability	10 (18.9)
	70-79	22 (41.5)			Anxiety	10 (18.9)
	80-89	20 (37.8)			Disinhibition	3 (5.6)
**Disease duration (years)**	≤7 years	36 (67.9)			Sadness	3 (5.6)
	>7 years	17 (32.1)		**MMSE (points)**	<13	17 (32.1)
**CDR (points)**	1	16 (30.2)			13-20	30 (56.6)
	2	28 (52.8)			>20	6 (11.3)
	3	9 (17)				

**Figure 1:**
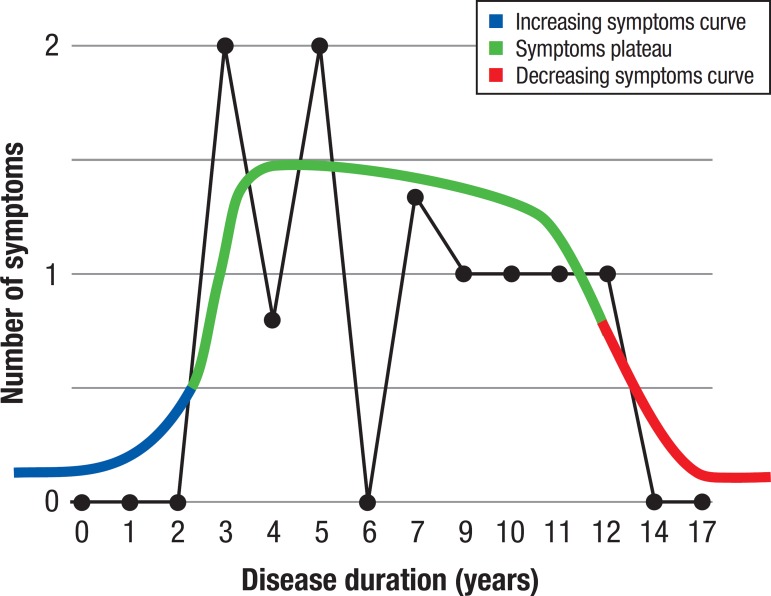
The Inverted-U theory. Black graph shows the distribution of the number of symptoms with disease duration. Initially, the number of symptoms increased with disease progression (Increasing Symptoms Curve), followed by a constant presentation of symptoms (Symptoms Plateau) and a decrease in symptoms at the advanced phase of dementia (Decreasing Symptoms Curve).

There was no significant relationship between disease duration and CDR score (p = 0.43). However, there was a negative association between disease duration and number of symptoms (OR = 0.083, 95%CI: 0.006-1.11; p = 0.034). On the graph plotted (Figure 1), the number of symptoms shows a tendency to increase in the initial years of dementia progression, remain relatively constant for some time, and then decrease at late stages.

There was an association between CDR scores of 1 or 2 points and the presence of neuropsychiatric symptoms (OR = 6.16, 95% CI: 1.36-27.9, p = 0.02).

A multiple ordinal regression was performed to determine whether CDR score was significantly correlated with number of NPS, adjusted for disease duration and dementia etiology. For this analysis, age, sex, MMSE score and number of symptoms were considered independent variables. CDR score was considered the dependent variable. CDR score remained significantly associated with number of NPS (p = 0.02) after adjusting for disease duration and etiology (MxD or vascular). Thus, a linear regression was conducted for each NPS, considered a dichotomized variable (present or absent), adjusted for the variables previously described, to assess whether any specific symptom had a large effect on the association with CDR score, but no significant correlation with individual symptoms was found.

## DISCUSSION

Apathy was the most prevalent NPS and this finding is consistent with previous studies.^6^^,^ 15 ^,^ 16 However, the prevalence of agitation and depression, which are significantly associated with VaD and MxD according to previous studies,^15^^,^ 16 was lower in our sample. In a study conducted by Siqueira-Neto et al. in southeast Brazil, the most prevalent disturbances were psychosis and hallucinations, which may be partially explained by the higher mean CDR scores in their sample and, subsequently, greater overall severity of dementia.^4^

The negative association between disease duration and number of symptoms seems to be related to the impairment of the neuroaffective circuits, due to progressive degeneration. Neurochemical studies have shown abnormal distribution of neurotransmitters, particularly in the basal forebrain cholinergic system, leading to widespread disconnection of cholinergic projections caused by ischemic events.^17^^-^ 19 These neuronal abnormalities seem to cumulate with time of disease evolution and reduce expression of symptoms derived from affected cortical areas.

Whereas some studies report that NPS prevalence, particularly apathy, increases over time,^20^^,^ 21 other suggest it remains stable^22^^,^ 23 or decreases.^24^ In the cited studies, however, the patients had lower CDR and MMSE baseline scores than the patients in our analysis. In addition, the studies involved shorter follow-up periods. This suggests that NPS may behave like an inverted "U" curve, increasing initially when dementia is less severe, maintaining a short Plateau with relatively constant NPS, and decreasing with later progression (advanced dementia) when severity is much greater. This theory is illustrated in Figure 1.

The results found are consistent with an aging population, especially considering cerebral and systemic vascular disease consequences. The NPS observed in this study sample can be explained not only by the dementia, but also by risk factors that interact with vascular load and neurodegenerative lesions, negatively impacting patient cognition,^25^ with etiology related to either acute or progressive disease.

One of the explanations for the negative relationship between disease duration and number of symptoms might be a positive response to treatment, given that one of the goals of neurodegenerative disease management is to increase quality of life through multidisciplinary neurorehabilitation.^21^^,^ 25

Although performed in a referral unit, the small sample is a limitation of the study, leading to potential bias in the data analysis. However, the negative association between duration and number of symptoms is coherent with neurophysiopathological findings and may be a key to better understanding the evolution of the disease and providing more focused treatment approaches. Further studies should be fostered to confirm these findings.
